# Postnatal prophylaxis and the use of presumptive HIV therapy for the prevention of vertical transmission of HIV in Canada 1997–2020

**DOI:** 10.1002/jia2.26510

**Published:** 2025-06-03

**Authors:** Jeanne Brochon, Terry Lee, Jason Brophy, Joel Singer, Marie‐Elaine Metras, Jeannette Comeau, Alena Tse‐Chang, Athena McConnell, Deborah Money, Isabelle Boucoiran, Laura J. Sauve, Ari Bitnun, Fatima Kakkar

**Affiliations:** ^1^ Division of Pediatric Infectious Department of Pediatrics CHU Sainte Justine, Université de Montréal Montréal Quebec Canada; ^2^ CIHR Pan‐Canadian Network for HIV & STBBI Clinical Trials Research Vancouver British Columbia Canada; ^3^ Children's Hospital of Eastern Ontario, University of Ottawa Ottawa Ontario Canada; ^4^ Pharmacy Department CHU Sainte Justine, Université de Montréal Montréal Quebec Canada; ^5^ IWK Health, Dalhousie University Halifax Nova Scotia Canada; ^6^ Stollery Children's Hospital, University of Alberta Edmonton Alberta Canada; ^7^ Jim Pattison Children's Hospital, University of Saskatchewan Saskatoon Saskatchewan Canada; ^8^ Women's Hospital and Health Centre of British Columbia, University of British Columbia Vancouver British Columbia Canada; ^9^ Division of Maternal‐Fetal Medicine Department of Obstetrics and Gynecology Université de Montréal Montreal Quebec Canada; ^10^ Women and Children Infectious Disease Center, Sainte‐Justine Research Center Montreal Quebec Canada; ^11^ School of Public Health, Université de Montréal Montreal Quebec Canada; ^12^ Hospital for Sick Children, University of Toronto Toronto Ontario Canada

**Keywords:** antiretroviral therapy, children, perinatal HIV, postnatal prophylaxis, risk factors, vertical transmission

## Abstract

**Introduction:**

Presumptive HIV therapy (PHT) is recommended for post‐natal HIV prophylaxis (PNP) in situations at high risk of HIV vertical transmission (VT), for both prevention of transmission and as early treatment in cases of in utero transmission. The objective of this study was to describe the risk of VT and use PHT among newborns in Canada, and specifically, factors associated with the use of PHT.

**Methods:**

Data were analysed for all mother‐infant pairs (MIPs) in the Canadian Perinatal HIV Surveillance Program (1997−2020), collected annually from 22 perinatal HIV centres in Canada. Infants were categorized as high risk (delivery viral load [dVL] ≥1000 copies/ml or maternal combined antiretroviral [cART] <4 weeks prior to delivery), moderate risk (dVL detectable and <1000 copies/ml, and maternal cART ≥4 weeks prior to delivery) and low risk (dVL undetectable and maternal cART ≥4 weeks prior to delivery). Neonatal prophylaxis and HIV transmission risk were compared between groups.

**Results:**

A total of 4743 MIPs were included in the analysis. Overall, 13.3% of newborns received PHT; the most prescribed PHT regimens included combinations using zidovudine, lamivudine and nelfinavir (48.5%) or nevirapine (41.9%). While the most significant risk factor for transmission on univariate analysis was a detectable dVL ≥1000 copies/ml versus undetectable (odds ratio [OR] 27.91 [11.20−69.54]), the risk remained significantly increased at dVL between 400 and 999 copies/ml (OR 31.71 [8.31−120.98], but not at dVL between 50 and 399 copies/ml (OR 3.03 [0.72−12.81]). At dVL 50–399 copies/ml, 29.8% of infants received PHT, increasing to 46.7% at dVL 400–999 copies/ml, and 64.4% of infants at dVL≥1000 copies/ml. The overall risk of transmission was 6% in the high‐risk group, 0.5% in the moderate‐risk group and 0.2% in the low‐risk group.

**Conclusions:**

PHT has been widely used in Canada in situations at high risk of VT, with 25% of newborns in this risk group receiving PHT as PNP. While PHT may reduce the risk of VT in high‐risk situations and may be of benefit in cases of VT, these data also highlight ongoing gaps in perinatal HIV prevention in Canada.

## 1 INTRODUCTION

Neonatal antiretroviral prophylaxis is an important component of the prevention of vertical transmission (VT) of HIV, with different regimens used globally according to the availability of formulations suitable for use in newborns and the estimated risk of VT. Annually, approximately 250–300 women living with HIV (WLHIV) give birth in Canada, where the overall risk of perinatal transmission is estimated at 1% [1, 2, 3]. Current guidelines for post‐natal prophylaxis (PNP) for Canadian newborns of WLWH are stratified based on HIV control during pregnancy [4, 5]. For infants born to WLWH who received < 4 weeks of combined antiretroviral (cART), have delivery viral load (dVL) ≥ 1000 copies/ml or are suspected of non‐adherence, enhanced postnatal prophylaxis, currently referred to as presumptive HIV therapy (PHT) with zidovudine (ZDV), lamivudine (3TC) and nevirapine (NVP) or raltegravir (RAL), is recommended for the infant for 4–6 weeks [6, 7, 8]. For all other infants, 4–6 weeks of oral ZDV recommended. For infants born to women who received ≥4 weeks of cART prior to delivery but have a low‐level detectable dVL (20−999 copies/ml), the use of PHT is not consistent across centres. This could reflect differences in provider perception of risk at this level of dVL, where there is limited data on transmission risk [9].

While PHT is generally well‐tolerated [10, 11], there are both immediate and potentially long‐term risks associated with its use. In the short‐term, these include anaemia, neutropenia, pancreatitis and hepatitis [12, 13], and though mostly reversible, can lead to premature discontinuation of treatment [14]. In the long term, there is concern regarding the potential cumulative impact of both in utero and post‐natal Antriretroviral therapy (ART) exposure on mitochondrial function and associated sequelae [15, 16]. In Canada, the use of PHT versus monotherapy is also associated with greater treatment complexity, as some of the medications used with a formulation suitable for infants (e.g. NVP suspension and RAL granules for suspension) require federal (Health Canada) Special Access Program approval on a case‐by‐case basis [17], and can be challenging to procure in remote settings and in a timely manner. The choice of the third drug in PHT regimens has, therefore, changed over time according to drug availability, safety record, site preference and maternal resistance history [14, 18]. The objective of this study was, therefore, to describe the risk of VT and use PHT among newborns in Canada, and specifically, factors associated with the use of PHT.

## 2 METHODS

### 2.1 Study design

This was a secondary analysis of a multicentre national study based on data prospectively collected by the Canadian Perinatal HIV Surveillance Program (CPHSP) from 1997 to 2020. While the CPHSP was initiated in 1991, the period from 1991 to 1996 was excluded from this analysis as no PNP was available during this time. The CPHSP includes 22 centres from across Canada involved in perinatal HIV care, and represents 95% of pregnant WHLH in Canada. Programme details, data captured and temporal trends have been previously published [1, 19, 20]. Data variables have been added to the programme over time, with dVL (from routine clinical testing) and adherence only added in 2007, and breastfeeding in 2020.

All children followed at one of the participating centres and born to a WLWH in Canada between 1997 and 2020 were included in the study, with the exception of children born to WLWH whose HIV status was diagnosed after the infant's birth. Children were considered not living with HIV if they had two or more negative virologic tests before 18 months of life, one of which was at least 4 weeks after PNP cessation; or a negative serology after 18–24 months of life. We defined dVL as the closest VL measured near delivery (within 4−6 weeks before delivery or up to 2 weeks after delivery if no prior VL was available). Undetectable dVL was defined as <50 copies/ml. Given that the sensitivity of VL testing changed over time and was heterogenous across centres, women who had dVL <20 copies/ml or whose dVL was “target not detected” were categorized as <50 copies/ml for the analyses.

Infants were classified into three groups based on perceived risk of VT as follows: high risk if maternal dVL was ≥ 1000 copies/ml, or if duration of maternal cART was <4 weeks before delivery; moderate risk if maternal dVL was detectable but < 1000 copies/ml and if maternal cART was initiated at least 4 weeks before delivery, and low risk if maternal dVL was undetectable, and if maternal cART was initiated at least 4 weeks before delivery. These risk groups were defined by our previous risk stratification in the CPHSP for annual reporting purposes [1, 2]. We categorized PNP as no treatment, monotherapy (ZDV or NVP), dual‐drug regimen, or PHT if the regimen consisted of three or more drugs. If two regimens were used, infants were analysed in the group corresponding to the highest number of drugs received. Adherence was defined as excellent (>95%), suboptimal (<95%) or absent when there was no or unknown ART, as determined by the site principal investigator upon chart review.

### 2.2 Ethics considerations

The CPHSP was approved by each centre's research ethics board (REB) when required locally, and nationally by UBC CREB HO7‐02384 as a public health surveillance programme. The requirement for individual patient consent has been waived by the REB for the purposes of the surveillance study.

### 2.3 Statistical analyses

Descriptive statistics were used to describe the use of PNP, and the chi‐square test of trend to compare neonatal PNP use across groups defined by maternal risk factors. To identify factors associated with VT, we conducted univariate analysis according to potential explanatory variables based on risk factors for VT identified a priori based on literature [13]. Given the limited number of VT events and the amount of missing data for some predictors, we did not conduct a multivariable analysis (adhering to the 10 events per variable rule), nor did we impute missing data as there were no good surrogate variables which were not missing.

## 3 RESULTS

A total of 4911 mother‐infant pairs (MIPs) were registered in the CPHSP between 1997 and 2020. After the exclusion of women who were diagnosed after delivery (*n* = 168), 4743 MIPs were included in the analysis (Table 1). There were 58 newborns with perinatally acquired HIV between 1997 and 2020. The most significant risk factor for transmission on univariate analysis was detectable dVL (≥1000 copies/ml vs. undetectable) (odds ratio [OR] 27.91 [11.20−69.54]). There was a significantly increased risk of VT among women with dVL between 400 and 999 copies/ml versus undetectable (OR 31.71 [8.31−120.98], but not among those with dVL between 50 and 399 copies/ml (OR 3.03 [0.72−12.81]). Other significant risk factors for VT included being diagnosed with HIV during pregnancy versus pre‐conception (OR 6.61 [3.77−11.59]), receiving <4 weeks treatment versus cART ≥4 weeks during pregnancy (OR 35.51 [16.20−82.26]), suboptimal versus excellent maternal adherence (OR 30.85 [9.74−97.78]) and injection drug use versus heterosexual sex as a mode of HIV acquisition (OR 2.16 [1.23−3.78]). Among cases where precise timing of infant testing was recorded, 1686/3174 (53.1%) of infants had birth PCR, confirming in utero infection in 14/3174 (0.44%) of newborns.

**Table 1:** Risk factors for vertical transmission in CPHSP cohort from 1997 to 2020

	No transmission *N* (%)	Transmission *N* (%)	*p*‐value	OR CI 95%	*p*‐value
**Total** ^a^	4583 (98.8)	58 (1.2)			
**Risk category**			0.046		
Heterosexual	3046 (99.0)	31 (1.0)		1.00	
Injection drug	919 (97.9)	20 (2.1)		2.16 [1.23−3.78]	0.007
Perinatal	77 (100.0)	0 (0.0)		0.62 [0.04−10.48]	0.743
Other	98 (100.0)	0 (0.0)		0.49 [0.03−8.19]	0.620
Unknown	443	7			
**Maternal dVL**			<0.001		
≥1000 c/ml	120 (90.2)	13 (9.8)		27.91 [11.20−69.54]	<0.001
Detectable‐999 c/ml	232 (97.9)	5 (2.1)		5.89 [1.94−17.89]	0.002
400−999	27 (90.0)	3 (10.0)		31.71 [8.31−120.98]	<0.001
50−399	205 (99.0)	2 (1.0)		3.03 [0.72−12.81]	0.132
Undetectable	1868 (99.6)	7 (0.4)		1.00	
Unknown	2363	33			
**Time of diagnosis**			<0.001		
Pre‐conception	3329 (99.4)	21 (0.6)		1.00	
During pregnancy	691 (96.0)	29 (4.0)		6.61 [3.77−11.59]	<0.001
	563	8			
**ART during pregnancy**			<0.001		
No ART	230 (89.1)	28 (10.9)		45.61 [22.19−93.73]	<0.001
Monotherapy	305 (98.4)	5 (1.6)		6.64 [2.35−18.78]	<0.001
cART <4 weeks	146 (91.2)	14 (8.8)		36.51 [16.20−82.26]	<0.001
cART ≥4 weeks	3873 (99.7)	10 (0.3)		1.00	
Unknown	29	1			
**Adherence**			<0.001		
Excellent	2218 (99.9)	3 (0.1)		1.00	
Suboptimal	359 (95.5)	17 (4.5)		30.85 [9.74−97.78]	<0.001
None	230 (89.1)	28 (10.9)		78.37 [25.60−239.90]	<0.001
Unknown	1776	10			
**Time period**			0.231		
1997−2014	3140 (98.6)	44 (1.4)		1.41 (0.78, 2.56)	0.258
2015−2020	1443 (99.0)	14 (1.0)		1.00	

Abbreviations: ART, antiretroviral therapy; cART, combined antiretroviral therapy; CI, confidence interval; dVL, viral load at delivery; OR, odds ratio.

^a^ Missing data are included for each variable as “unknown,” and percentages were calculated over the total in each category with available information.

### 3.1 Neonatal PNP

Table 2 describes the use of neonatal PNP. Overall, 13.3% of newborns received PHT, 14.5% a dual‐drug regimen and 70.4% monotherapy. When maternal dVL was detectable but between 50 and 399 copies/ml, 29.8% of newborns received PHT. This increased to 46.7% of newborns of women with dVL between 400 and 999 copies/ml, and 64.4% of newborns of women with dVL ≥1000 copies/ml. Overall, the most common maternal factors leading to the use of PHT included diagnosis during pregnancy (31%), detectable dVL (26%), followed by receipt of <4 weeks cART (25%).

**Table 2:** PNP use among newborns of WLWH in CPHSP cohort from 1997 to 2020

	No ART *N* (%)	Monotherapy *N* (%)	Dual ART *N* (%)	PHT *N* (%)	*p*‐value
**Total** ^a^	86 (1.8)	3310 (70.4)	682 (14.5)	627 (13.3)	
**Maternal factors**					
**Ethnicity**					
Black	27 (1.1)	1640 (68.7)	379 (15.9)	342 (14.3)	<0.001
Indigenous	25 (2.5)	731 (72.0)	153 (15.1)	106 (10.4)	
White	26 (2.7)	657 (69.0)	124 (13.0)	146 (15.3)	
Other	2 (0.7)	240 (82.8)	22 (7.6)	26 (8.9)	
Unknown	6	42	4	7	
**Maternal infection type**					
Perinatal	1 (1.3)	47 (60.3)	9 (11.5)	21 (26.9)	<0.001
Injection drug	31 (3.3)	659 (69.0)	154 (16.1)	111 (11.6)	
Heterosexual	43 (1.3)	2135 (68.6)	484 (15.6)	450 (14.5)	
Other	1 (0.9)	92 (91.1)	3 (3.0)	5 (5.0)	
Unknown	10	337	32	40	
**Time of diagnosis**					
Prior to conception	40 (1.2)	2515 (73.9)	446 (13.1)	400 (11.8)	<0.001
During pregnancy	17 (2.3)	379 (51.9)	141 (19.3)	194 (26.5)	
Unknown	29	416	95	33	
**ART during pregnancy**					
No treatment	46 (17.1)	76 (28.3)	62 (23.0)	85 (31.6)	<0.001
Monotherapy	8 (2.6)	227 (72.5)	62 (19.8)	16 (5.1)	
cART <4 weeks	2 (1.2)	62 (38.3)	27 (16.7)	71 (43.8)	
cART ≥4 weeks	30 (0.8)	2931 (74.5)	526 (13.4)	446 (11.3)	
Unknown	0	14	5	9	
**Adherence**					
Excellent	16 (0.7)	1820 (80.7)	328 (14.6)	90 (4.0)	<0.001
Suboptimal	6 (1.6)	172 (45.3)	67 (17.6)	135 (35.5)	
None	46 (17.1)	76 (28.3)	62 (23.0)	85 (31.6)	
Unknown	18	1242	225	317	
**Maternal VL at delivery**					
Undetectable (<50)	15 (0.8)	1459 (76.5)	318 (16.7)	116 (6.0)	<0.001
50−399	3 (1.4)	114 (54.8)	29 (13.9)	62 (29.8)	
400−999	0 (0.0)	11 (36.7)	5 (16.7)	14 (46.7)	
≥ 1000 c/ml	2 (1.5)	18 (13.3)	28 (20.8)	87 (64.4)	
Unknown	66	1708	302	348	
**Time period**					<0.001
1997−2014	65 (2.0)	2110 (65.9)	573 (17.9)	452 (14.1)	
2015−2020	21 (1.4)	1200 (79.7)	109 (7.2)	175 (11.6)	

Abbreviations: ART, antiretroviral therapy; cART, combined antiretroviral therapy; PHT, presumptive HIV therapy; VL, viral load.

^a^ Missing data are included for each variable as “unknown,” and percentages were calculated over the total in each category with available information.

Among 4743 MIPs in the total cohort, 2891 had sufficient data for risk categorization (Table 3). The prevalence ratio of VT was 6% (49/813) in the high‐risk group, 0.5% (1/196) in the moderate‐risk group and 0.2% (5/1882) in the low‐risk group. The most common regimens used in the low‐risk group were ZDV monotherapy (76%), followed by a dual‐drug regimen of ZDV+3TC (15%), followed by combinations of PHT (6%). In the moderate‐risk group, the majority (55%) of infants received ZDV monotherapy, while 16% received a dual‐drug regimen, and 26% PHT. In the high‐risk group, 46% had received ZDV monotherapy, 19% a dual‐drug regimen and 25% PHT. The most prescribed PHT across all risk groups included AZT+3TC+nelfinavir (48.5%), followed by ZDV+3TC+NVP (41.9%), followed by combinations including four or more drugs (6.4%).

**Table 3:** Neonatal drug regimens and transmission by risk group in CPHSP cohort from 1997 to 2020

	Total cohort^a^ *N* (%)	High risk *N* (%)	Moderate risk *N* (%)	Low risk *N* (%)
**Total**	4743	813 (28.1%)	196 (6.7%)	1882 (65%)
**Transmissions**	58 (1.2%)	49 (6%)	1 (0.5%)	5 (0.2%)
Unknown	102	24	5	44
**PNP**				
None	86 (1.8)	57 (7.1)	2 (1.0)	14 (0.7)
Monotherapy				
ZDV	3299 (99.7)	374 (99.7)	109 (100.0)	1425 (99.5)
Single‐dose NVP	4 (0.1)	1 (0.03)	0 (0.0)	2 (0.1)
Others	7 (0.2)	0 (0.0)	0 (0.0)	5 (0.5)
Dual				
ZDV + 3TC	408 (59.8)	57 (34.1)	15 (50.0)	286 (90.8)
ZDV + NVP	253 (37.1)	101 (60.5)	15 (50.0)	23 (7.3)
ZDV + DDI	1 (0.2)	0 (0.0)	0 (0.0)	0 (0.0)
ZDV + LPV/r	1 (0.2)	0 (0.0)	0 (0.0)	1 (0.3)
Others	19 (2.7)	9 (5.4)	0	5 (1.6)
PHT				
ZDV + 3TC + NFV	304 (48.5)	45 (21.8)	9 (17.7)	39 (35.8)
ZDV + 3TC + NVP	263 (41.9)	128 (61.8)	35 (68.6)	61 (56.9)
ZDV + 3TC + RAL	13 (2.1)	2 (1.0)	5 (9.8)	6 (5.5)
ZDV + 2 others	4 (0.6)	3 (1.4)	0 (0.0)	0 (0.0)
Other triple ART	3 (0.5)	2 (1.0)	0 (0.0)	0 (0.0)
≥4 ART	40 (6.4)	27 (13.0)	2 (3.9)	2 (1.8)
Unknown	38	7	4	13
**Duration**				
<4 weeks	77 (1.8)	12 (1.9)	5 (2.8)	29 (1.6)
≥4 weeks	4221 (98.2)	628 (98.1)	172 (97.2)	1742 (98.4)
Unknown	359	116	17	97

Abbreviations: ART, antiretroviral therapy; DDI, didanosine; LPV/r, lopinavir/ritonavir; NFV, nelfinavir; NVP, nevirapine; PHT, presumptive HIV therapy; PNP, post‐natal prophylaxis; 3TC, lamivudine.

^a^ Risk group was unknown for 1852 infants.

Overall, among cases where VT occurred and there was sufficient data for risk stratification (*n* = 55), 21% of newborns had not received any PNP, 14% monotherapy, 12% a dual‐drug regimen and 53% had received PHT. Among the five newborns with VT in the low‐risk group, four had received PHT, due to difficulties with adherence in pregnancy (*n* = 2) and third‐trimester viraemia (*n* = 2). Birth PCR was available for four of the five newborns and was positive in two. In the single newborn with VT in the moderate‐risk group, PHT was used, birth PCR was not done, but positive at 2 weeks of age. Among the 49 newborns with VT in the high‐risk group, 51% (25/49) had received PHT; of them, 12/17 (70%) with a birth HIV PCR were positive confirming in utero HIV acquisition. Overall, where the timing of diagnostic PCR was clearly documented (37/55 cases), 46% were identified through birth PCR, 24% at 2 weeks of age, 20% between 1 and 2 months of age and 10% at 4 months of age.

## 4 DISCUSSION

In this study, we describe the risk of VT and the use of PHT in Canada through the CPHSP. Canada was one of the first countries to adopt PHT for newborns at high risk of VT, and with increasing interest in its use globally, our findings highlight some of the potential benefits and challenges of this approach.

Overall, the identified risk factors for VT in our cohort are similar to previously published findings from other cohorts, including late maternal diagnosis, maternal ART duration < 4 weeks before delivery, poor antenatal adherence to treatment and detectable dVL [21, 22, 23]. At low levels of maternal dVL, we observed a significantly increased risk among women with dVL 400–999 copies/ml versus undetectable (OR 31.71 [8.31−120.98], but not among those with dVL 50–399 copies/ml (OR 3.03 [0.72−12.81]). While we may have been underpowered for statistical significance at this low dVL threshold, our findings are similar to those reported by the French perinatal cohort, which showed a very low transmission rate when dVL was between 50 and 400 copies/ml of 0.2% (95% CI [0.01−1.10]) [24]. In our cohort, PHT was used in 29.8% of newborns with low‐level dVL (50−399 copies/ml) and 46.7% of cases at dVL 400–999 copies/ml, reflecting heterogenous practice in Canada at this level of dVL.

Of concern, 1.8% of all infants received no PNP, and 35.6% of infants born to women with dVL≥1000 copies/ml did not receive PHT. While this may be due to a lack of awareness of current guidelines by physicians, site‐specific preferences and drug availability, it may also be that these high‐risk exposures were not known to physicians in real time, as data from the CPHSP is entered retrospectively, and birth dVL may not have been known at the time of the PNP prescriptions. Moreover, PHT was also given to newborns not categorized as high risk, including 6% of those classified as low risk. This may in part reflect the changing threshold for viral load detection, with some sites able to capture VL <50 copies/ml. As the risk of transmission in this category is unknown, this may have resulted in increasing numbers of low‐risk infants prescribed PHT. It is also likely that factors other than dVL and duration of maternal ART were considered by physicians in the decision to use PHT, demonstrated by the use of PHT among 4/5 low‐risk cases where VT occurred. These cases highlight one of the key challenges in establishing guidelines and algorithms to capture the risk of in utero infection for the purposes of early treatment, as reliance on dVL and duration of ART may not be sufficient to identify all infants at risk of in utero infection, who may benefit from PHT [25, 26, 27].

Our study has several limitations inherent to the retrospective use of surveillance data. First, data collection through surveillance is limited, and as such, we do not have key variables such as the exact timing of maternal diagnosis, compliance with prescribed treatments, maternal drug resistance profiles, and most importantly, VL trajectory during pregnancy. We, therefore, could not capture the estimated risk of in utero transmission and how that might have influenced the decision to use PHT. We are also limited by a significant amount of missing data and a small number of VT events, limiting out ability to study the association between maternal ART regimen and transmission, and conduct multivariable analyses. Moreover, birth PCR was only done or documented in 53.1% of newborns; as such, we could not confirm the timing of infection (in utero vs. intrapartum) for the full cohort and address the question of the usefulness of PHT for prevention.

## 5 CONCLUSIONS

Our findings indicate that one in eight Canadian newborns of WLWH was prescribed PHT for PNP, resulting in 63% of infants acquiring HIV benefiting from early treatment. While PHT may be helpful in reducing the risk of VT in high‐risk situations and may be of benefit in cases of VT, these data suggest that interventions designed at improving engagement in care during pregnancy remain of paramount importance.

## COMPETING INTERESTS

FK and IB received career awards from Fonds de Recherche Santé Québec. The authors declare the following conflicts: FK research support from Altona and honoraria from ViV. IB: Research support from Moderna, Altona.

## AUTHORS’ CONTRIBUTIONS

TL performed, and JB contributed to, the data analysis. JB and FK conceptualized the study and protocol. JB drafted the manuscript, and TL, JB, JS, M‐EM, JC, AT‐C, AM, DM, IB, LJS, AB and FK revised the manuscript critically for important intellectual content. All of the authors contributed to data interpretation and gave final approval of the version to be published and agreed to be accountable for all aspects of the work.

## FUNDING

No specific funding was secured for this study. The HIV/AIDS Surveillance Section, Surveillance and Risk Assessment Division of the Public Health Agency of Canada provides funding for the Canadian Perinatal HIV Surveillance Program. The CIHR Canadian HIV Trials Network provides data management and statistical support for the Canadian Perinatal HIV Surveillance Program.

## Data Availability

The data that support the findings of this study are available on request from the corresponding author. The data are not publicly available due to privacy and ethical restrictions.
